# The frequency of Achilles and plantar calcaneal spurs in gout patients

**DOI:** 10.3906/sag-2011-201

**Published:** 2021-08-30

**Authors:** Emine DURAN, Emre BİLGİN, Ali İhsan ERTENLİ, Umut KALYONCU

**Affiliations:** 1 Division of Rheumatology, Department of Internal Medicine, Medical School of Hacettepe University, Ankara Turkey

**Keywords:** Gout, Achilles tendon, bone spurs, comorbidity

## Abstract

**Background/aim:**

Gout may cause various radiographic abnormalities such as cartilage loss, spurs, sclerosis, and periostal new bone formation. The purpose of this study was to investigate the frequency of Achilles and plantar spurs and related factors in gout patients.

**Matherial and methods:**

We performed a retrospective review of gout patients, treated at Hacettepe University hospitals between 2014 and 2019. We identified patients from the hospital records using the ICD-10 code (M10). Demographic and clinical features, comorbidities, and foot radiographies were collected. The radiographies were evaluated by a rheumatologist (U.K.) who was experienced in musculoskeletal radiography. Factors predicting the spurs were analyzed by logistic regression analysis.

**Results:**

181 patients who had lateral foot radiograph were included in this study. Eighty-one (44.7%) patients had score ≥ 2 Achilles spur, 81 (44.7%) patients had score ≥ 2 plantar spur, and 22 (12.1%) patients had no spur. Age, disease duration, duration between the gout diagnosis and appearing spur, the presence of metabolic comorbidities and hypertension were higher in both Achilles and plantar spurs than no spur group. Forty (22.1%) patients had score ≥ 2 both Achilles and plantar spur. In this group, the mean age was older and the proportion of metabolic comorbidities was higher than the groups of Achilles and plantar spur with a score 0 or 1. Predictor of the development of large or moderate-severe calcaneal spur was the existence of metabolic comorbidity [OR (95% CI): 3.49 (1.11–11.0) and p = 0.033].

**Conclusion:**

The presence of metabolic comorbidities increases the frequency of calcaneal spurs in gout patients. This condition can be explained by the impaired microvascular structure and increased hypoxia resulting in calcification on the tendon and ligament insertion sites.

## 1. Introduction

Gout is an inflammatory arthritis caused by the chronic deposition of monosodium urate (MSU) crystals. Crystal depositions can occur in the joints and soft tissues, leading to the inflammatory response [1]. A search for crystals in synovial fluid is recommended in patients with suspected gout, because MSU crystals provide a definite diagnosis of gout. When crystal identification is not possible, demonstration of atypical MSU crystal deposition (double contour sign and tophi) by ultrasound is very important for diagnosis [2]. Imaging studies have presented that 25% to 40% of individuals with asymptomatic hyperuricemia have MSU crystal deposition [3,4]. MSU crystals frequently deposit within joints and periarticular structures, especially at the first metatarsophalangeal joint, midfoot and knee [5]. Soft tissue structures such as tendons and ligaments are often influenced by MSU crystal deposition in gout patients [6]. The Achilles tendon is the most frequently affected tendon (39.1%) by this accumulation, followed by the peroneal tendons (18.1%) and other tendons are rarely involved [7]. These observations suggest that biomechanical strain or tissue stress may contribute to deposition of MSU crystals [8].

Soluble intracellular uric acid due to longstanding hyperuricemia could cause the endothelium dysfunction [9]. Impaired tissue oxygenation due to damaged microvascular structure increases the frequency of Achilles tendinopathy [10]. When not adequately treated, gout may cause in radiographic abnormalities, such as cartilage loss, punched-out erosions, sclerotic overhanging edges, and spur [11]. Calcaneal spurs are bony outgrowths on the tendinous and ligamentous attachments of the calcaneus. The common sites for spur formation are the insertion of the Achilles tendon on the posterior margin of the calcaneus and the attachment of the plantar aponeurosis on the inferior surface of the calcaneus [12]. Calcaneal spurs are a typical cause of heel pain that show to rise with age [13]. The frequency of Achilles and plantar spurs in gout patients is not known.

The aim of our retrospective study was to investigate the frequency of Achilles and plantar spurs and related factors in patients with gout.

## 2. Materials and methods

### 2.1. Study population

In this retrospective cohort study, we included the patients with gout who were followed-up in our department from January 2014 to March 2019. Patients with gout were identified from the hospital electronic medical records, by using the International Classification of Diseases (ICD)-10 code for gout and other crystal arthritis (M10, M11). The code for other crystal arthritis was included in order not to miss gout patients who were recorded wrongly with M11. All the demographic and clinical features and follow-up data were obtained from the patient files. Gout diagnosis was confirmed by combining patients’ history, clinical presentation, laboratory tests during gout attacks and between attacks, status regarding the prescription of colchicine and uric acid lowering drugs. 

### 2.2. Data collection 

We collected the following demographic data: age (at the time of foot radiograph was taken), sex, body mass index (BMI; at the time of radiographic examination was done), and metabolic comorbidities (hypertension, diabetes mellitus, dyslipidemia, coronary heart disease, and chronic renal disease).

Regarding gout; disease duration, duration between the gout diagnosis and radiographic evaluation, mean serum uric acid level before the lateral foot radiography, usage of colchicine, uric acid lowering drug, and glucocorticoid, and radiographic scores of the spurs were recorded. 

The foot radiography of the patients were evaluated in terms of Achilles and plantar calcaneal spurs by a rheumatologist (UK) who experienced in musculoskeletal radiography. Achilles spurs were graded as absent (score = 0), small (score = 1), and large (score = 2, 3, and 4). Plantar calcaneal spurs were scored as absent (score = 0), small (score = 1), moderate (score = 2) or severe (score = 3) using standard radiologic atlas images. Both Achilles spurs and plantar calcaneal spurs were dichotomized as being absent-small (score 0 or 1) or large/moderate-severe (score 2 or 3 for plantar spurs; score 2, 3, and 4 for achilles spurs) [13,14]. The patients who had Achilles or plantar calcaneal spurs were compared accordingly.

### 2.3. Statistical analyses

Statistical analysis was performed using the Statistical Package for the Social Sciences software (v: 25.0; IBM Corporation, Armonk, NY, USA). The variables were investigated using visual (histogram, probability plots) and analytic methods (Kolmogorov–Smirnov, skewness and curtosis) to determine whether they were normally distributed or not. The data of descriptive analysis were expressed as either mean ± standard deviation (SD) or the median, interquartile range (IQR). Categorical variables were compared with the Chi-square test or Fisher’s exact test where appropriate. The Student-t test and Mann–Whitney U test was used to compare the normal and nonnormally distributed continuous data between two groups, respectively. 

To find risk factors associated with the development of large/moderate-severe Achilles and plantar spurs, score ≥ 2 (patients with both Achilles and plantar spur score ≥2) vs. score 0 and/or 1 were compared. The variables with p < 0.1 as a result of univariate analysis were included in the multivariate analysis. Factors predicting the presence of calcaneal spurs were analyzed by the logistic regression analysis. Hosmer–Lemeshow goodness-of-fit statistics were used to assess model fit. Among the variables with strong intragroup correlations that were considered representing the whole cohort were included in the analysis. If any significant interactions were found, results were given by stratification. A 5% type-I error level was used to infer statistical significance.

## 3. Results

### 3.1. Study population and patient characteristics

**Figure 1:**
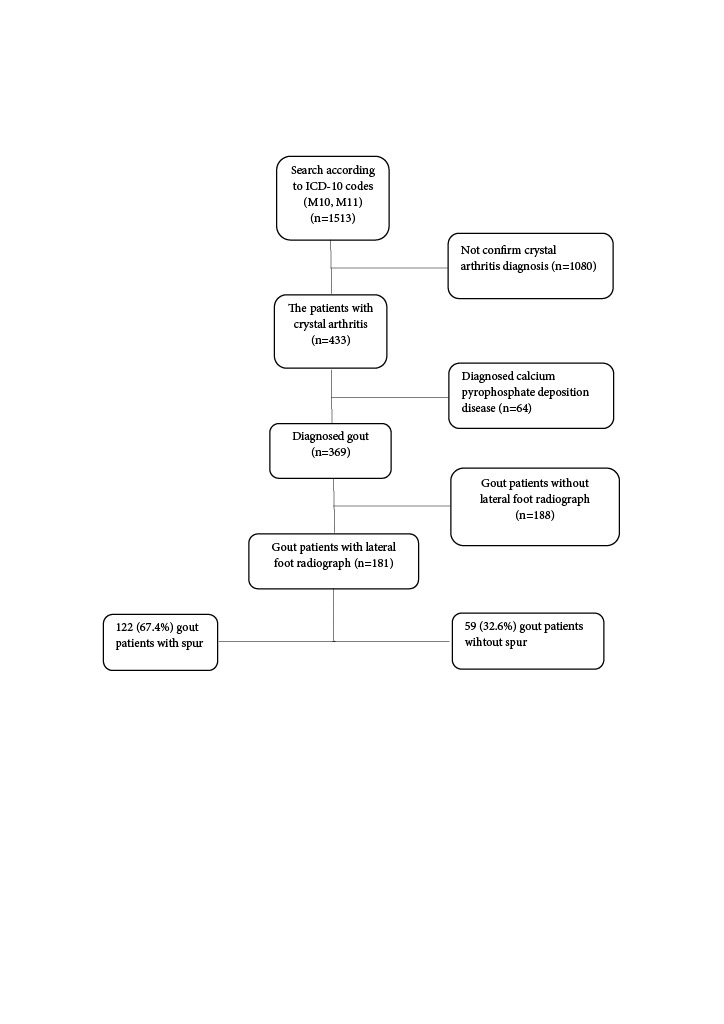
Flowchart of the patients enrollment.

Search according to ICD-10 codes (M10, M11) revealed a total of 1513 patients. Of these patients, 433 were diagnosed with crystal arthritis according to the physician’s decision. Crystal arthritis other than gout and gout patients without lateral foot radiograph were excluded (Figure 1).

A total of 181 patients (49%) who had lateral foot radiograph among 369 gout patients were included in the study. Mean age was 58.2 ± 14.6 years and 147 (81.2%) of patients were male. Median disease duration was 5.1 (4.5) years. Mean uric acid level before the foot radiograph was 8.5 ± 1.8. Metabolic comorbidities were present in 107 (59.1%) gout patients, and the most common comorbidity was hypertension in 87 patients (48.1%). Details were given in Table 1. 

**Table 1:** Comparison of variables score ≥ 2 Achilles spurs, score ≥ 2 plantar spurs, and no spur.

Variables*	All patients(n = 181)	Achilles spurScore ≥ 2(n = 81)	Plantar spurScore ≥ 2(n = 81)	No spur(n = 22)	p*	p†
Age, years (mean ± SD)	58.2 ± 14.6	60.5 ± 12.8	63.4 ± 11.7	48.2 ± 17.2	0.004	0.001
Male	147 (81.2)	63 (77.8)	55 (67.9)	20 (90.9)	0.17	0.03
Disease duration, years (median, IQR)	5.1 (4.5)	5.3 (5.1)	5 (4.4)	3.9 (3.8)	0.002	0.008
Duration between gout diagnosis and spur,years (med, IQR)		4.4 (6)	3.6 (6.4)			
Uric asid level before the radiograph (mg/dL)(mean ± SD)	8.5 ± 1.8	8.2 ± 1.8	8.3 ± 1.8	8.9 ± 1.8	0.09	0.17
BMI (kg/m2) (mean ± SD)	28.7 ± 4	29.4 ± 4.3	28.6 ± 3.9	27.9 ± 3.8	0.19	0.51
BMI ≥ 30	53 (29.3)	26 (36.6)	21 (31.3)	6 (33.3)	0.89	0.96
Existence metabolic comorbidity	107 (59.1)	56 (69.1)	58 (71.6)	8 (36.4)	0.005	0.002
- Hypertension	87 (48.1)	44 (54.3)	51 (63)	5 (22.7)	0.009	0.001
- Diabetes mellitus	26 (14.4)	14 (17.3)	17 (21)	2 (9.1)	0.35	0.2
- Dislipidemia	20 (11)	8 (9.9)	10 (12.3)	3 (13.6)	0.61	0.87
- Cardiovascular disease	49 (27.1)	22 (27.2)	18 (22.2)	7 (31.8)	0.67	0.35
- Chronic renal disease	20 (11)	11 (13.6)	11 (13.6)	2 (9.1)	0.57	0.57
Overall medical treatment						
- Colchicine	180 (99.4)	81 (100)	80 (98.8)	22 (100)	0.99	0.99
- Urat lowering drug	164 (90.6)	73 (90.1)	76 (93.8)	18 (81.8)	0.28	0.09
- Glucocorticoid	77 (42.5)	37 (45.7)	38 (46.9)	7 (31.8)	0.24	0.2

### 3.2. Distribution of Achilles and plantar spurs

**Figure 2:**
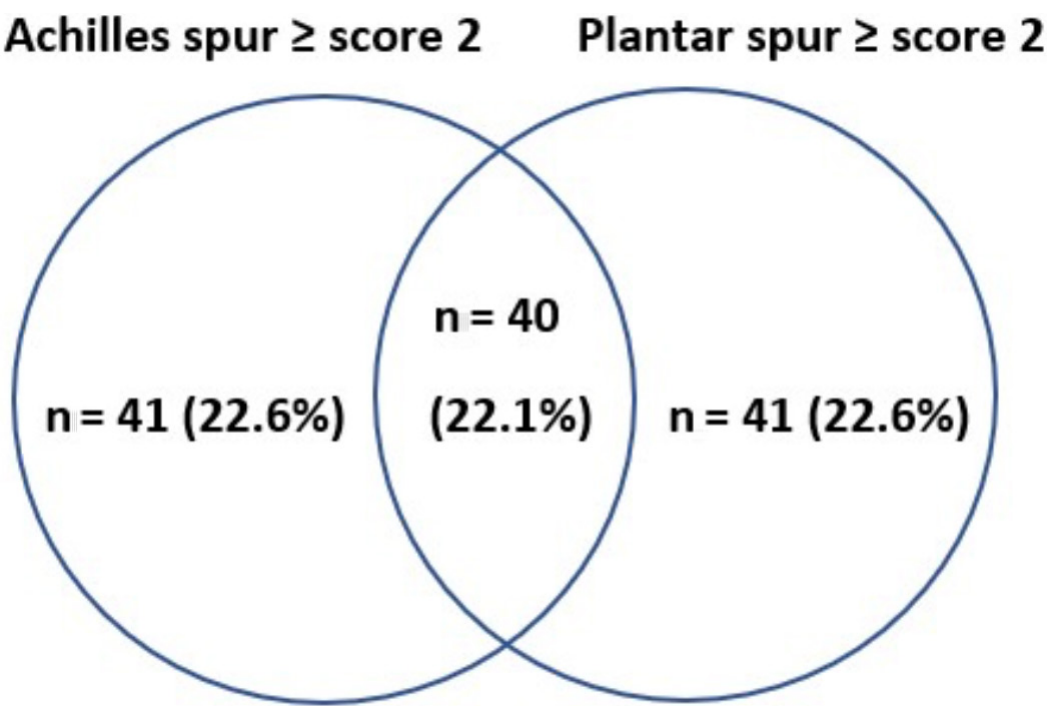
The cluster of patients with spurs scoring at least 2.

On the lateral foot radiographs, 81 (44.7%) of patients had at least score-2 Achilles spur, 81 (44.7%) of patients had at least score-2 plantar spur, and 40 (22.1%) patients had score ≥2 Achilles and plantar spur (Figure 2). Age, disease duration, the ratio of metabolic comorbidities, and hypertension were higher in Achilles spur group compared to no spur group. These results were similar to the comparison of patients with score ≥ 2 plantar spurs vs. no spur group (Table 1).

**Figure 3:**
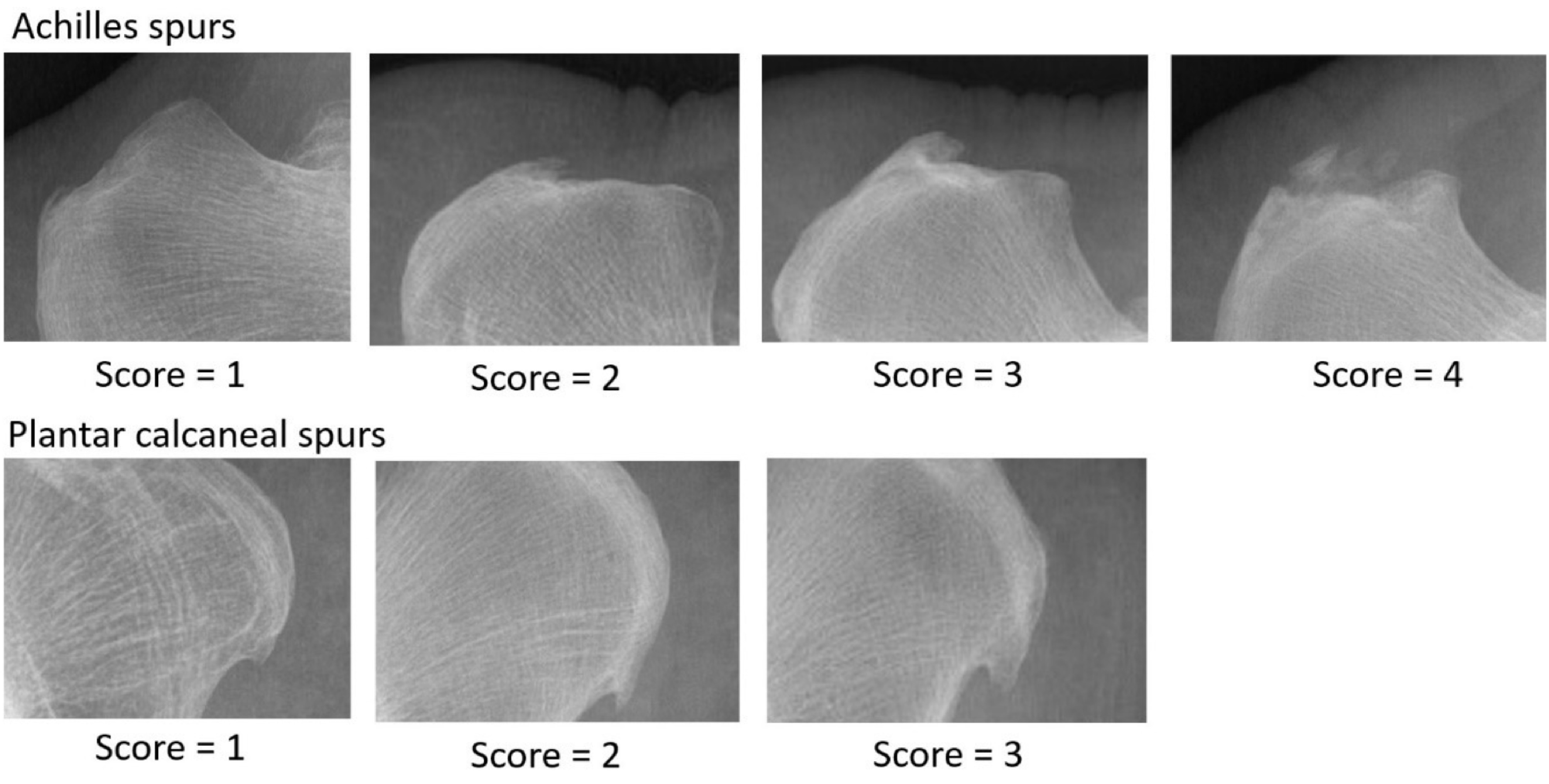
Typical examples of the different scores of Achilles and plantar calcaneal spurs.

Typical examples of the different classes of spurs were shown in Figure 3. Achilles spur rates were as follows: 38 (22%) absent, 84 (46.4%) score 1, 45 (24.9%) score 2, 31 (17.9%) score 3, and 12 (6.6%) score 4 of all gout patients, respectively. According on whether the Achilles spur was unilateral or bilateral: 33 (23.1%) unilateral and 104 (77%) bilateral. For plantar spurs; 65 (37.6%) absent, 41 (22.6%) score 1, 43 (23.7%) score 2, and 47 (26%) score 3 of all gout patients, respectively. According on whether the plantar spur was unilateral or bilateral: 31 (26.7%) unilateral and 77 (66.4%) bilateral (Table 2). 

**Table 2:** The scores of Achilles spurs and plantar calcaneal spurs.

Grading of spur (n = 173*)								
Right	Score 0 (n,%)- Achilles- Plantar	38 (22)65 (37.6)	9 (5.2)4 (2.3)		03 (1.7)			
	Score 1- Achilles- Plantar	20 (11.6)12 (6.9)	27 (15.6)14 (8.1)		12 (6.9)3 (1.7)	4 (2.3)1 (0.6)		
	Score 2- Achilles- Plantar	4 (2.3)11 (6.4)	10 (5.8)7 (4)		14 (8.1)8 (4.6)	3 (1.7)7 (4)		1 (0.6)
	Score 3- Achilles- Plantar	01 (0.6)	2 (1.2)0		1 (0.6)4 (2.3)	19 (10.1)33 (19.1)		1 (0.6)
	Score 4- Achilles					1 (0.6)		9 (5.2)
	Score	Score 0	Score 1		Score 2	Score 3		Score 4
	L e f t							
	Total unilateral- Achilles- Plantar	33 (23.1)31 (26.7)		Total bilateral- Achilles- Plantar			104 (70.6)77 (66.4)	

### 3.3. Risk factors for the development of Achilles and plantar spurs

Forty (22.1%) of patients had score ≥ 2 both Achilles and plantar spur. In this group, the mean age was older than the groups of Achilles and plantar spur with score 0 or 1 (mean (SD); 63.6 (±11.1)/55.3 (±17.4), p = 0.01). The ratio of males and mean uric acid level before the radiograph were significantly lower in the group of score ≥ 2 Achilles and plantar spur. The proportion of existence metabolic comorbidity and hypertension was higher in this group (Table 3). To find risk factors associated with the development of large/moderate-severe Achilles and plantar spurs, we performed a multivariate analysis. Enter method was used for final model. A quantitative interaction was found between sex and metabolic morbidity, so, regression models were performed by stratifying the cohort according to sex. For females, no relevant parameter was found to predict large or moderete-severe spurs. In male patients, multivariate analysis adjusted for age (at the time of lateral foot radiograph was taken) and mean serum uric acid level before the radiograph demonstrated that predictors of large or moderate-severe spurs are existence metabolic comorbidity [OR (95% CI): 3.49 (1.11–11.0) and p = 0.033] (Table 4).

**Table 3:** Comparison of variables among Achilles and plantar spurs at least score 2 vs. score 0 or 1.

Variables*	Achilles andplantar spur (at least score 2)(n = 40)	Achilles and plantar spur (score 0 or 1)(n = 40)	p
Age, years (mean ± SD)	63.6 ± 11.1	55.3 ± 17.4	0.01
Male	26 (65)	37 (92.5)	0.003
Disease duration, years (med, IQR)	5 (5)	4.3 (4.5)	0.2
Duration between the gout diagnosis and spur, years (median, IQR)	4.4 (6)	3.6 (6.4)	0.86
Uric asid level before the radiograph (mg/dL) (mean ± SD)	7.9 ± 1.7	8.7 ± 1.6	0.02
BMI (kg/m2)(mean ± SD)	29 ± 4	28.1 ± 3.8	0.35
BMI ≥ 30	12 (35.3)	11 (32.4)	0.65
Existence metabolic comorbidity	30 (75)	20 (50)	0.02
- Hypertension	26 (65)	14 (35)	0.007
- Diabetes mellitus	9 (22.5)	4 (10)	0.13
- Dislipidemia	2 (5)	6 (15)	0.13
- Cardiovascular disease	7 (17.5)	15 (37.5)	0.05
- Chronic renal disease	5 (12.5)	3 (7.5)	0.45

**Table 4:** Multivariable linear regression analyses to find the predictor factors of large/moderate-severe Achilles and plantar spurs.

Variables	OR	95% CI	p
Male			
- Age at the radiograph	1.01	0.98–1.05	0.408
- Mean uric acid level before the radyograph	0.92	0.72–1.18	0.530
- Presence of metabolic comorbidity	3.49	1.11–11.0	0.033
Female			
- Age at the radiograph	1.01	0.94–1.09	0.624
- Mean uric acid level before the radyograph	0.74	0.48–1.13	0.171
- Presence of metabolic comorbidity	0.18	0.02–1.38	0.688

## 4. Discussion

In this study, we reported the frequency of Achilles and plantar calcaneal spur in gout patients. Both Achilles and plantar calcaneal spurs were common in our cohort. One hundred and twenty-two (67.4%) patients had a score ≥ 2 Achilles or plantar spurs. To the best of our knowledge, there is no information in the literature regarding the frequency of calceneal spur in gout patients. 

MSU accumulation near a joint is associated with spurs, periosteal new bone formation, ankylosis, and particularly osteosclerosis and osteophytosis [11]. Conventional radiography remains the primary imaging tool in the workup of these conditions. The spur is identified as a sharp spicule of dense bone proliferation extending at an acute angle from the cortex [15]. Although spur formation is a well-known, the connection between these spurs and their prevalence with age and sex remain are variable [16]. Toumi et al. showed that plantar and Achilles spurs were highly prevalent in older people and there was a moderate positive correlation between Achilles and plantar spurs for women <30 years of age but not for males [13]. In another study, the incidence of both plantar calcaneal and Achilles spurs increased by age and Achilles spur was significantly more common in females, while there was no difference in the incidence of plantar calcaneal spurs between males and females [17]. Similarly, in our study, the average age of the patients with Achilles spur and plantar spur score ≥2 was older than the group without any spur. Additionally, gout disease duration and duration between the gout diagnosis and appearing spur were longer in these patients. However, these differences were not shown in multivariate analysis for predictor factor in patients with Achilles and plantar spur score ≥2. 

Calcaneal spurs have been reported in almost all types of arthritis including gout [12]. In a controlled study, in patients with rheumatoid arthritis, calcaneal spurs occurred with a slightly higher frequency than in normal subjects (21% vs. 16%) [18]. In another controlled study, calcaneal spurs were more common in subjects with psoriatic arthritis than controls [19]. Alltough the frequency of calcaneal spurs in gout patients is unknown, irregular calcifications or spurs around the joint have been demonstrated [20]. In a recent study involving 530 participants, the rate of plantar calcaneal spur was 26.5% in patients with plantar heel pain [14]. Toumi et al. showed that the prevalence of Achilles or plantar spurs in general population was 38% [13]. However, in both studies, no information was given about the rheumatic diseases or metabolic comorbidities of the patients. In our research, the rate of large Achilles spur and moderate-severe plantar spur was 44.7%, separately. This superior rate may be explained by our patients were diagnosed with gout and had the high rate of metabolic comorbidity.

Gout is both an inflammatory and a metabolic disease. Prospective cohorts have shown that gout and hyperuricaemia associated with hypertension, renal failure, type 2 diabetes mellitus (DM) and metabolic syndrome [21–24]. Hyperuricemia activates the renin-angiotensin system, decreases nitric oxide synthase activity, stimulates vascular smooth muscle cell proliferation, and promotes insulin resistance. In addition, soluble intracellular uric acid could damage the endothelium and arteries [9]. In our cohort, the mean uric acid levels were 8.5 mg/dL (±1.8) and the rate of metabolic comorbidities was ~60%. This high rate of metabolic comorbidities means that when gout is diagnosed then comorbidities need to be considered and managed appropriately. 

Association between the presence of calcaneal spurs and self-reported comorbidities were explored in a study. In contrast to our study, there was no significant difference between the presence and absence of calcaneal spur in 198 patients with self-reported co-morbidities (diabetes mellitus, stroke, peripheral vascular disease, and hypertension) [16]. In another study comparing of risk factors and comorbidities in patients with and without plantar calcaneal spurs, the rate of diabetes mellitus was found more frequently in patients with spur [25]. In our cohort, the proportion of large or moderate-severe calcaneal spur was higher in gout patients with metabolic comorbidity. 

On the other hand, diffuse idiopathic skeletal hyperostosis (DISH) which is characterized by entheseal ossification and bone spurs is associated with several metabolic derangements and concomitant diseases including obesity, hypertension, dyslipidaemia, DM, hyperuricaemia, and an increased risk for cardiovascular diseases [26]. Similarly, in our study, the percentage of metabolic comorbidities was found higher in patients with spurs. 

One of the major limitations of our study was including no control group. Absence of a control group can keep from determining whether other causes such as metabolic comorbidities other than gout contribute to heel spurs. Another main limitation was the lack of a valid method of foot X-ray taken to evaluate spurs and missing information about the patients’ drug adherence due to retrospective study. Another limitation was the difference the time from diagnosis of gout to foot radiography in patients with and without spur. Also, selection bias for the study may have created that the frequency of calcaneal spur was common in our cohort because of the radiography was more commonly applied to gout patients with heel pain.

Our results demonstrate that the presence of metabolic comorbidity in gout patients may be an indicator of calcaneal spurs. This can be explained by the impaired microvascular structure, damaged endothelium, and increased hypoxia resulting in calcification on the tendon and ligament insertion sites. 

## Informed consent

Our study is compliant with the Helsinki Declaration and approved by Hacettepe University ethical committee (Approval number: GO 19/304).
